# A Homologous Bacterin Protects Sheep against Abortion Induced by a Hypervirulent *Campylobacter jejuni* Clone

**DOI:** 10.3390/vaccines8040662

**Published:** 2020-11-06

**Authors:** Zuowei Wu, Michael J. Yaeger, Orhan Sahin, Changyun Xu, Ashenafi F. Beyi, Paul J. Plummer, Melda Meral Ocal, Qijing Zhang

**Affiliations:** 1 Veterinary Microbiology and Preventive Medicine, Iowa State University, Ames, IA 50010, USA; wuzw@iastate.edu (Z.W.); cyxu@iastate.edu (C.X.); afbeyi@iastate.edu (A.F.B.); pplummer@iastate.edu (P.J.P.); meralmelda@hotmail.com (M.M.O.); 2 Veterinary Pathology, Iowa State University, Ames, IA 50010, USA; myaeger@iastate.edu; 3 Veterinary Diagnostic and Production Animal Medicine, Iowa State University, Ames, IA 50010, USA; osahin@iastate.edu; 4 National Institute of Antimicrobial Resistance Research and Education, Ames, IA 50010, USA

**Keywords:** sheep abortion, *Campylobacter jejuni*, clone SA, bacterin, immunoprotection

## Abstract

*Campylobacter jejuni* clone SA has emerged as the predominant cause of ovine abortion outbreaks in the United States (US). Despite the fact that commercial *Campylobacter* vaccines are available, their efficacy in protecting abortion induced by *C. jejuni* clone SA is uncertain, and a protective vaccine is needed to control the disease. In this study, an experimental homologous bacterin (made of a clone SA isolate) and two commercial *Campylobacter* vaccines were evaluated for their protection against *C. jejuni* clone SA-induced sheep abortion. All vaccines induced high levels of antibodies against *C. jejuni* clone SA in pregnant ewes, but only the experimental homologous bacterin produced significant protection (80%). Immunoblotting showed that the experimental vaccine elicited more specific antibodies against *C. jejuni* clone SA. These findings strongly suggest the necessity of developing a homologous vaccine for the control *C. jejuni* clone SA induced abortion on sheep farms.

## 1. Introduction

Abortion in ewes causes significant economic loss to sheep producers [1,2,3]. *Campylobacter* infection is one of the most prevalent causes of ovine abortion in The United States (US) and worldwide, with an overall abortion rate of 5% to 50% in affected flocks [4,5]. Historically, *C. fetus* subsp. *fetus* (herein referred to as *C. fetus*) accounted for the majority of *Campylobacter* species associated with ovine abortion; however, since the 1990s, the major species associated with the disease has been replaced by *C. jejuni* in the US [6,7]. Particularly, a hypervirulent *C. jejuni* clone (ST-8; named clone SA for sheep abortion) emerged in the US and has become the predominant *Campylobacter* agent (>90%) of sheep abortion [7]. The *C. jejuni* SA clone is abortifacient and is present in the intestinal tract of healthy ruminant animals [8]; however, in pregnant ewes, it translocates across the intestinal barrier and spreads via blood to the placenta and uterus, where it multiplies and induces abortion [7]. Clinically, *Campylobacter*-induced sheep abortion typically occurs in the last trimester of pregnancy [4]. Pathologically, microscopic placental lesions consist of septal necrosis, leukocyte infiltration, and high numbers of bacteria within subtrophoblastic capillaries and the chorionic villus stroma, while aborted or stillborn fetuses may have no gross lesions or they may show subcutaneous sero-sanguineous edema and liver lesions [9]. On culture, high numbers of *Campylobacter* can be recovered from the aborted placentas, fetal stomach contents, and, to a lesser extent, lung and liver of aborted lambs [7,9]. In addition to its high pathogenicity, *C. jejuni* clone SA is resistant to tetracycline, the only class of antibiotics approved for control and prevention of *Campylobacter* abortion in sheep in the US [7], making the control of sheep abortion even more difficult.

It has been documented that vaccination is effective in preventing ovine campylobacteriosis [10,11,12]. However, experimental studies revealed inadequate cross-protection against infections by heterologous serotypes or even inadequate protection against homologous challenges in ewes vaccinated with commercial vaccines [11,13]. Moreover, *Campylobacter* spp.-associated abortions occurred in flocks that received commercial monovalent and bivalent vaccines [6,14], indicating that individual vaccines may not be effective against all *Campylobacter* strains due to genetic and antigenic variations among different strains. In the US, there are currently two commercially available ovine-labeled polyvalent *Campylobacter* vaccines: one supplied by Colorado Serum Co. (Denver, CO, USA) and the other one manufactured by Hygieia Laboratories (Woodland, CA, USA). Both vaccines are bacterins, and each contains a mixture of *C. fetus* and *C. jejuni* strains. In a previous study using guinea pigs that were immunized with the two commercial vaccines and an experimental bacterin made of *C. jejuni* clone SA, the vaccines produced varied protection against *C. jejuni* clone SA-induced bacteremia. However, protection against abortion was not evaluated in the study due to the lack of pregnancy in majority of the guinea pigs [15]. Thus, whether the commercial vaccines and the experimental bacterin can protect sheep from *C. jejuni* clone SA-induced clinical abortion remains to be determined.

Given the high prevalence and predominant role of *C. jejuni* clone SA in causing sheep abortion in the US [7], it is imperative to develop a protective vaccine and assess the efficacy of commercial vaccines against this highly pathogenic strain in its natural host. For this purpose, we performed immunization of pregnant ewes with an experimental bacterin made of *C. jejuni* clone SA in comparison with the two commercially available vaccines. The immunized animals were challenged with *C. jejuni* clone SA to evaluate the efficacy of the vaccines in protection against clinical abortion.

## 2. Materials and Methods

### 2.1. Animals

Timed bred pregnant ewes (whiteface and black–whiteface crosses) were sourced from Iowa State University Sheep Teaching farm at an average fetal age of 58 days. Ewes were confirmed pregnant via ultrasound and were kept in the facility in four groups (three vaccine groups and one sham group), and individual ewes were identified with ear tags. A total of 10 ewes were enrolled for each vaccine group, and nine ewes were enrolled for the sham group. Immediately after grouping, ewes were vaccinated at the teaching farm as described in the vaccination regimen below. After immunization, the ewes were then moved to the Laboratory Animal Resources (LAR) facility at Iowa State University for *Campylobacter* challenge. All ewes were fed antibiotic-free Teklad-Envigo 7060 small ruminant complete ration and water ad libitum. All procedures undertaken were approved by the Institutional Animal Care and Use Committee at Iowa State University (IACUC-18-134).

### 2.2. Vaccination

The experimental homologous bacterin was prepared using *C. jejuni* clone SA strain IA3902 and adjuvant aluminum hydroxide gel (Accurate Chemical & Scientific Co. Westbury, NY, USA) as described previously [15]. The approximate density of bacterial cells was 6.7 × 10^8^ colony-forming units (CFU)/mL for the experimental bacterin [15]. A sham vaccine was prepared by mixing the adjuvant aluminum hydroxide gel in sterile phosphate-buffered saline (PBS) solution (0.2 mg/mL). Two commercial polyvalent vaccines labeled for prevention of campylobacteriosis in sheep were also included in the study. Each of the commercial vaccines contained a mixture of multiple *C. fetus* and *C. jejuni* strains in an aluminum hydroxide adjuvant. One was manufactured by Colorado Serum Co. (Denver, CO, USA) (designated vaccine A in this study) and the other one was produced by Hygieia Laboratories (Woodland, CA, USA) (designated vaccine B in this study). The vaccines were administered subcutaneously in the interscapular region at approximately mid-gestation (50–100 days), and the doses were 5 mL for vaccine A, 2 mL for vaccine B, 4 mL for the experimental homologous bacterin, and 4 mL for the sham vaccine. The dose amount and number for commercial vaccine A and B were suggested by the product instructions. Two doses of each vaccine were administered 2 weeks apart. All ewes were bled prior to the first dose and 2 weeks post the second dose for serology. After vaccination, the animals were observed regularly, and none of the vaccines induced clinical signs or adverse reactions.

### 2.3. Intravenous Challenge and Necropsy

For each animal, the upper half of the left jugular furrow was shaved, and the area was aseptically prepared using alternate Chlorhexidine^®^ (chlorhexidine gluconate 2.0%) and isopropyl alcohol (70%) wipes. An 18G, 2 inch intravenous (IV) catheter was placed into the left jugular vein. Through the catheter, 1.25 mL of Banamine^®^ Solution (Flunixin Meglumine, Merck) was administered first to relieve potential inflammatory reaction, and then 1.25 mL of *C. jejuni* IA3902 inoculum (OD_600_ of 0.5) containing ~2 × 10^9^ live CFU was administered immediately, followed by 1 mL of sterile PBS to insure all of the inoculum was delivered. Animals were monitored twice daily for signs of illness including depression, loss of appetite, prolonged recumbency, elevated temperatures, and evidence of abortion (vaginal bleeding or expelled fetus). Ewes that became markedly depressed and recumbent by endotoxemia were euthanized for humane reasons. Ewes that exhibited vaginal bleeding or abortion were immediately euthanized and necropsied. The study was concluded at 18 days post inoculation, and all remaining animals were euthanized and necropsied at that time. At the necropsy, gross lesions of the uterus and placenta were examined, and postmortem samples were collected for *Campylobacter* culture and histopathology, including ewe heart blood, uterus, placenta, and fetal lung and liver tissue. Tissues for histopathology were placed in 10% neutral buffered formalin for 24 h, then transferred to 70% alcohol, trimmed, embedded in paraffin, and processed routinely for hematoxylin and eosin (H&E) staining. *Campylobacter* culture for heart blood and utero-placental tissues was performed by using *Campylobacter*-selective Mueller–Hinton (MH) agar plates as described previously [15]. Briefly, 250 µL of heart blood was plated for each animal, and utero-placental tissues were minced with scissors, swabbed with a sterile cotton swab, and streaked onto the *Campylobacter*-selective MH agar plates. *Campylobacter* colonies were confirmed to be clone SA by PCR amplifying the specific gene CJSA_1356 [16].

### 2.4. ELISA and Western Blotting

ELISA was performed to determine the level of *C. jejuni*-specific immunoglobulin G (IgG) antibodies in sheep sera as described previously [15]. Briefly, microtiter plates were coated with whole membrane components (approximately 60 ng/well) of *C. jejuni* clone SA strain IA3902. Serum samples were diluted in the blocking buffer to 1:100, while polyclonal, horseradish peroxidase-labeled rabbit anti-sheep IgG (ABCAM, Cambridge, MA, USA) was diluted to 1:1000 in the blocking buffer. The horseradish peroxidase substrate (ABTS 2-Component Microwell Peroxidase Substrate, Seracare Life Sciences, Milford, MA, USA) was used for developing the color. Optical density (OD) values of individual wells were measured using an ELISA reader (FLUOstar Omega Microplate Reader, BMG Labtech Inc., Durham, NC, USA) at the absorbance of 405 nm. For Western blotting, whole-membrane components (approximately 100 μg) of *C. jejuni* clone SA strain IA3902 were separated by SDS-PAGE using a one-IPG-well gel on Mini-PROTEAN^®^ Vertical Electrophoresis system (Bio-Rad, Hercules, CA, USA) and transferred onto polyvinylidene fluoride (PVDF) membranes as described previously [17,18]. The PVDF membrane was then cut into 3 mm wide strips. The 1:100 diluted sheep sera and 1:1000 diluted polyclonal, horseradish peroxidase-labeled rabbit anti-sheep IgG were used to detect reaction of sheep sera to individual antigenic bands on the strip blots. The strip blots were incubated with diluted sheep sera overnight at 4 °C and with rabbit anti-sheep IgG for one hour at 25 °C. The TMB Membrane Peroxidase Substrate System (Seracare Life Sciences, Milford, MA, USA) was used for band visualization [17].

### 2.5. Statistical Analysis

A commercial statistical software GraphPad Prism (GraphPad, San Diego, CA, USA) was used to perform all analyses. One-way ANOVA was used to detect differences in ELISA results obtained from sera. A value of *p* ≤ 0.05 was considered significant. A log-rank (Mantel–Cox) test was used to evaluate the difference of protection rate, and a value of *p* ≤ 0.01 was considered significant.

## 3. Results

### 3.1. All Bacterin Vaccines Elicited Antibody Responses against C. jejuni Clone SA

**Figure 1:**
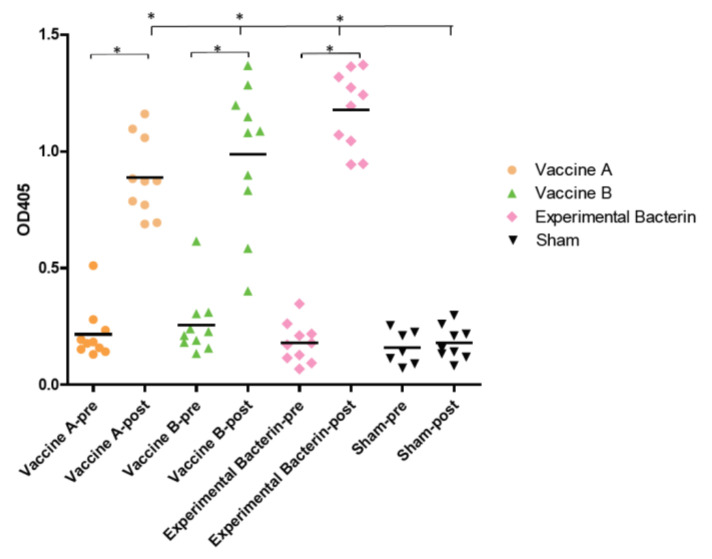
Vaccine-induced antibody levels against *Campylobacter jejuni* clone SA as determined by ELISA. Pre-vaccination sera (pre) and post-vaccination sera (post, 2 weeks after the last dose) for each group are plotted side by side. Each symbol represents the optical density (OD) value of a single animal. The bars represent the mean OD values of the groups. The mean ELISA OD values for post-vaccination sera immunized by vaccine A, vaccine B, and the experimental bacterin are significantly greater than those from the sham group and those from the pre-vaccination sera in each group (* *p* < 0.05).

**Figure 2:**
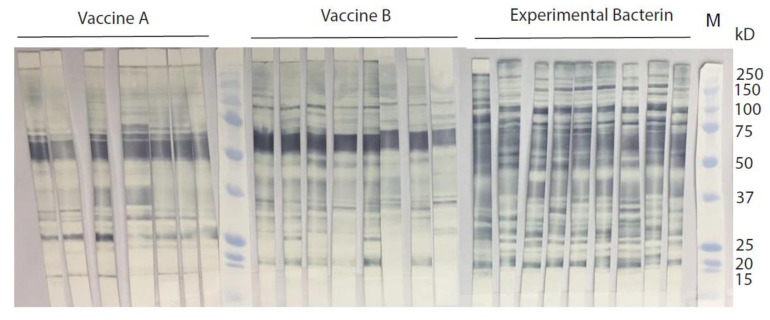
SDS-PAGE and immunoblotting of post-vaccination sera against *C. jejuni* clone SA antigens. The whole-membrane protein fraction of *C. jejuni* clone SA strain IA3902 was separated by SDS-PAGE and blotted with individual post-vaccination sera. Each strip represents the blotting result of a single serum sample. M indicates prestained protein standards (Bio-Rad, Hercules, CA, USA), and the sizes of the protein markers are indicated on the right.

Serum samples were collected from individual animals before and after vaccination to evaluate the antibody response to the whole-membrane antigens of *C. jejuni* clone SA by ELISA. As shown in Figure 1, the mean ELISA OD values of post-vaccinated sera were 0.884 for vaccine A (0.216 for the pre-vaccinated sera), 0.988 for vaccine B (0.256 for the pre-vaccinated sera), and 1.177 for the experimental bacterin (0.179 for the pre-vaccinated sera). For the sham group, the mean ELISA OD values were 0.159 (pre vaccination) and 0.180 (post vaccination), respectively. Statistical analysis showed that the ELISA OD values were significantly elevated (one-way ANOVA; *p* < 0.05) in the post-vaccinated sera than in the pre-vaccinated sera of each of the vaccinated groups, but there was no significant difference between the pre- and post-vaccinated sera in the sham control group. The ELISA results showed that both the two commercial vaccines and the experimental bacterin successfully induced high level of antibodies against *C. jejuni* clone SA. Although the mean ELISA OD value of the post-vaccinated sera in the experimental bacterin group was higher than those in the groups with vaccine A and B, only the difference between the experimental bacterin group and vaccine A group was statistically significant (one-way ANOVA; *p* < 0.05). To further evaluate the antibody profiles against clone SA, the post-vaccinated sera from each group were immunoblotted against the whole-membrane fraction of strain IA3902. Although all three vaccines elicited strong antibody responses in sheep (Figure 1), their antibody reaction patterns were significantly different as revealed by SDS-PAGE and Western blotting (Figure 2). It was visually apparent that the sera from animals vaccinated with the experimental bacterin recognized more antigenic bands on the blot compared with those sera vaccinated with the commercial bacterins.

### 3.2. The Experimental Homologous Bacterin Elicited High Immune Protection against Clone SA-Induced Abortion

**Figure 3:**
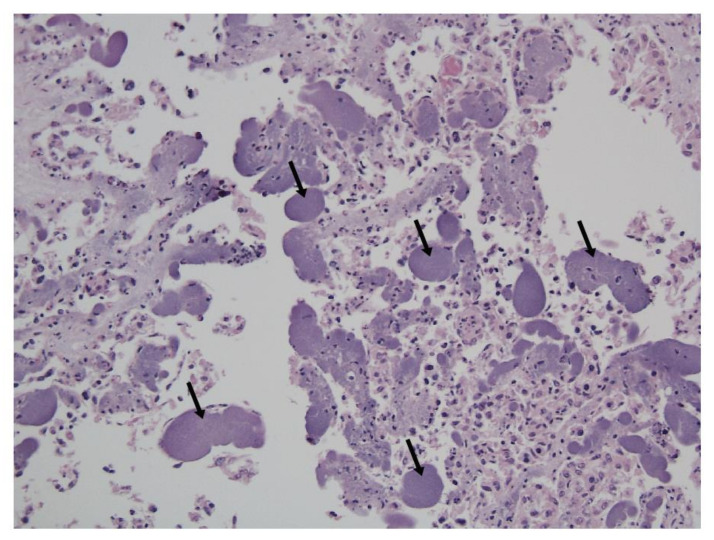
A representative hematoxylin and eosin (H&E) stain of placenta tissue with the subtrophoblastic capillaries distended by large numbers of intracytoplasmic bacteria typical of *Campylobacter* (arrows).

**Figure 4:**
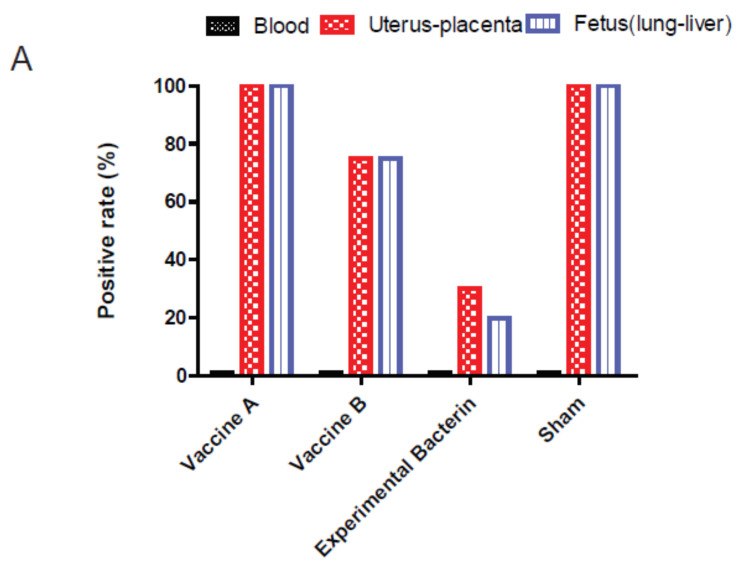

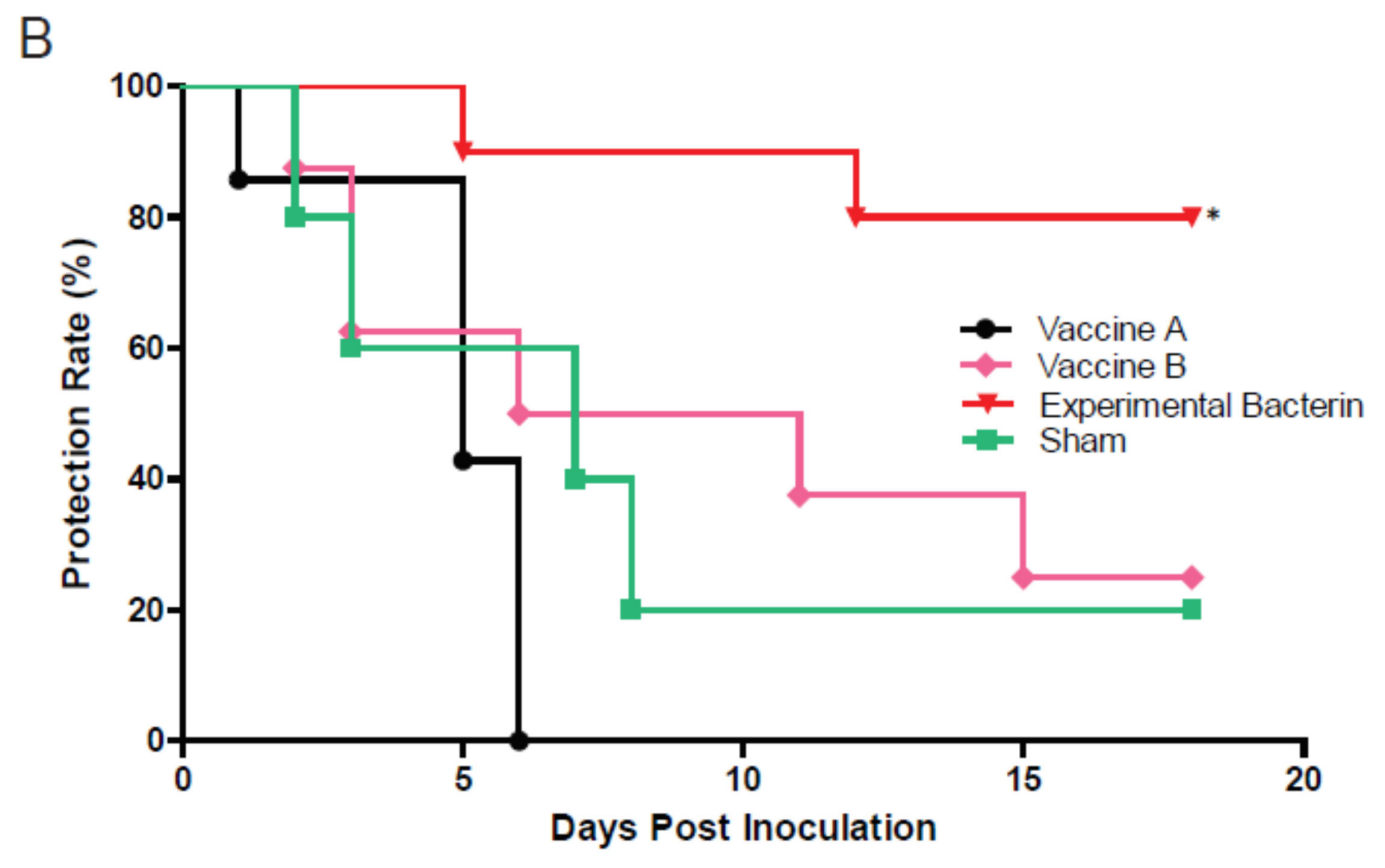
The efficacy of different vaccines in protecting sheep from *C. jejuni* clone SA-induced abortion. (**A**) *Campylobacter*-culture-positive rate in postmortem samples (ewe heart blood, utero-placenta, and fetal lung and liver tissue) at necropsy. (**B**) Protection rate induced by various vaccines during an 18 day observation period post challenge. The protection efficacy of the experimental bacterin vaccine is significantly different from the sham control (indicated by an asterisk; log-rank (Mantel–Cox) test; *p* < 0.01).

Immunized ewes were challenged intravenously by clone SA strain IA3902 to evaluate the protection efficacies of the bacterins. Although Banamine^®^ Solution was administered to relieve the acute inflammatory reaction induced by IV inoculation of *C. jejuni*, three out of 10 ewes from the vaccine A group, two out of 10 ewes from the vaccine B group, and three out of nine ewes from the sham control group developed acute septic shock with clinical signs of endotoxemia 1–2 days post challenge. The symptoms included fever (104–104.6 °F), anorexia, diarrhea, hyperpnea, depression, and recumbency accompanied by vaginal bleeding or abortion. These animals were euthanized when these clinical signs occurred, which were considered as endotoxin-induced abortion, and excluded from the efficacy analysis. However, notably, no ewes in the experimental bacterin immunized group developed endotoxemia. In addition, although the animal pregnancy was examined by ultrasound before the immunization, one from the sham group was found to be nonpregnant at the terminal necropsy and was excluded for the efficacy evaluation as well. Eventually, only the pregnant ewes without clinical signs of endotoxemia were included in the evaluation of the protection efficacy. These included seven ewes from the vaccine A group, eight ewes from the vaccine B group, 10 ewes from the experimental bacterin group, and five ewes from the sham control group. During the 3 weeks post challenge, we observed vaginal bleeding or abortion in 100% (7/7) of ewes in the vaccine A group, 75% (6/8) of ewes in the vaccine B group, 20% (2/10) of ewes in the experimental bacterin group, and 80% (4/5) of ewes in the sham group. Histologically, the aborted animals developed inflammation in the utero-placental tissues, and chorionic villus capillaries were loaded with bacterial cells (*Campylobacter*) (Figure 3). These histologic changes were absent in the placentas of *Campylobacter*-culture-negative and nonaborted ewes. All the aborted sheep were cultured positive with *Campylobacter* in the utero-placental tissue and the fetal lung and liver tissue (Figure 4A). Interestingly, no ewes were *Campylobacter*-positive in the heart blood at the time of necropsy, indicating that bacteremia is transient in the pathogenesis of abortion for clone SA. At the termination of the experiment, one ewe in the experimental bacterin group and one in the sham group were *Campylobacter*-positive in the utero-placental tissue via culture but did not have any histopathological changes in placental/uterine tissues and did not abort, which suggests that they were infected but had not yet developed clinical abortion. Therefore, they were not counted as abortion in their respective groups. Only ewes aborting and showing histopathological evidence of utero-placental *Campylobacter* infection were defined as no protection. As a result, the protection rate was 0% (0/7) for the vaccine A group, 25% (2/8) for the vaccine B group, 80% (8/10) for the experimental bacterin group, and 20% (1/5) for the sham group (Figure 4B). The protection rate of ewes immunized by experimental bacterin was significantly higher than that of the sham ewes (log-rank (Mantel–Cox) test, *p* < 0.01), but there was no significant difference in the protection rate between vaccines A and B groups and the sham group. The significantly lower abortion rate in the experimental bacterin group indicated that the homologous vaccine is highly effective in protecting sheep against *C. jejuni* clone SA-induced abortion.

## 4. Discussion

Vaccination plays a vital role in protecting animals from infectious diseases and is often used by sheep producers for the management of sheep abortion [5]. Due to the constant emergence of new pathogenic variants, it is important to examine the efficacy of existing and newly developed vaccines against the prevalent pathotypes. In this study, we evaluated an experimental bacterin in comparison with two commercially available vaccines in terms of their efficacy against *C. jejuni* clone SA, which is responsible for more than 90% of *Campylobacter*-induced sheep abortion in the US [7]. We found that the experimental homologous vaccine is highly effective in protecting sheep against *C. jejuni* clone SA-induced abortion, but the protection from the commercial vaccines is limited.

Under natural infection, the pathogenesis of abortion induced by *C. jejuni* entails oral exposure, intestinal colonization, bacterial invasion, bacteremia, and infection of the fetoplacental unit [19]. Ideally, *Campylobacter* vaccine efficacy should be evaluated by the end disease (utero-placental infection and subsequent abortion) following the oral infection route. However, there are several issues which have complicated experimental reproduction of this disease in sheep. Firstly, unlike many laboratory animal species, specific pathogen-free sheep are difficult to obtain. Secondly, in commercial populations, there is a high rate of *Campylobacter* carriage in the ovine gall bladder (42%), intestine (63.8%), and feces (75.0%) [20,21], leading to high rates of *Campylobacter* sero-positivity. Therefore, an oral challenge model to reflect the natural route of infection is currently not available. In our previous studies, we tried to induce utero-placental infections and abortion in sheep derived farms via oral, intraperitoneal (IP), and IV challenge with clone SA strain, but we only succeeded via IV challenge (unpublished data). However, *Campylobacter*, as a Gram-negative bacterium, contains lipooligosaccharides (LOS) in their outer membrane. When *Campylobacter* is injected directly into the blood, the LOS may cause uncontrolled activation of immune systems with production of inflammatory mediators [22] and lead to septic shock [23]. To reduce the septic shock triggered by the inoculum, we washed the cells with sterile PBS to minimize the inoculum-carried LOS and administered Banamine^®^ Solution to relieve the inflammatory reaction, but endotoxic shock still occurred in some of the ewes from the Vaccine A group, the Vaccine B group, and the sham group. However, no toxin shock symptoms were observed from the experimental homologous bacterin immunized group, suggesting that the homologous vaccine even conferred protection against endotoxic shock. In *Campylobacter*, LOS consists of a highly variable outer core of nonrepeating oligosaccharides anchored to lipid A [24], presenting highly variable antigens in different strains. The highly variable LOS represents a strategy for *Campylobacter* to evade host immune defenses [25]. It is possible that the commercial bacterins have different LOS structures from clone SA and, consequently, the antibody against LOS elicited by commercial vaccines may not be cross-reactive with the LOS of clone SA, resulting in the clinical differences in toxin shock.

In this study, abortion following the IV challenge was used as the evaluation endpoint to assess the efficacy of the *Campylobacter* vaccines. The results showed that the protection was considerably higher in animals that received the homologous bacterin (80%) than in those that received vaccine A (0%), vaccine B (25%), or a sham vaccination (20%), indicating that the experimental homologous vaccine is efficacious, while the commercial vaccines are not effective against *C. jejuni* clone SA-induced abortion (Figure 4). The commercial vaccines were made of a mixture of multiple *C. fetus* and *C. jejuni* strains, but it is unknown if clone SA was included at all or in sufficient quantity in the formulation. The ELISA results showed that both the commercial vaccines and the experimental bacterin elicited a high level of antibodies against clone SA (Figure 1), but Western blotting revealed a significant difference in the reactive patterns of the antibodies (Figure 2). The results suggest that, although the commercial vaccines induced antibodies cross-reactive with antigens in *C. jejuni* clone SA, the antibodies may not target the important virulence factors in *C. jejuni* clone SA or may not have sufficient amount to neutralize the virulence factors. Although the nature of protective antigens is unknown, the ELISA result and the protection outcome suggest that the commercial vaccines may not have sufficient protective antigens against *C. jejuni* clone SA, underscoring the need for a homologous vaccine to protect ewes from the currently prevalent and abortifacient *C. jejuni* strain (clone SA).

In a previous study using a guinea pig model, administration of vaccine B and the experimental bacterin made of *C. jejuni* clone SA significantly reduced bacteremia of clone SA following IP inoculation, while administration of vaccine A was ineffective despite the fact that it induced *Campylobacter*-specific antibody responses [15]. The findings from both the guinea pig model and the sheep challenge in this study consistently indicate that vaccine A is not protective at all against *C. jejuni* clone SA. However, the results on vaccine B were inconsistent between the guinea pig study and the sheep immunization. The pathogenic steps in naturally occurring sheep abortion involves a bacteremia stage, but the stage may be transient. In the guinea pig study, examination of bacteremia at 48 h following intraperitoneal (IP) inoculation might have missed the bacteremia peak in some animals due to the variations among individual animals, leading to the inaccurate evaluation of protective efficacy. On the other hand, the IV challenge used in this sheep study might have allowed more *Campylobacter* cells to get into the bloodstream instantaneously than the IP challenge, which overwhelmed the protection conferred by vaccine B and resulted in the subsequent utero-placental infection and abortion. According to the results from the guinea pig trial and the sheep study, it is reasonable to conclude vaccine B produced a low level of protection, but it was not sufficient to protect an IV challenge. In contrast to Vaccines A and B, the experimental vaccine consistently produced an adequate protection, indicating its suitability for prevention and control of sheep abortion in the US.

In conclusion, the efficacy of an experimental homologous bacterin and two commercial *Campylobacter* vaccines was evaluated against sheep abortion induced by the hypervirulent *C. jejuni* clone SA in this study. Although all vaccines induced high antibody levels against *C. jejuni* clone SA, only the experimental bacterin produced a robust protection against abortion induced by *C. jejuni* clone SA. Given that *C. jejuni* clone SA has become the predominant cause of sheep abortion in the US during the last decade and is highly resistant to tetracycline, an effective vaccine against this clone is critically needed for sheep producers to control the disease. This may be achieved by development of a homologous vaccine against *C. jejuni* clone SA or by modifying the existing vaccines to enhance their cross-protection against the abortion-inducing *C. jejuni* clone on sheep farms.
